# 

**Published:** 1890-01

**Authors:** 


### LIST OF CONTRIBUTORS TO VOLUME XI.

Angle, Edward H., D.D.S.
Atkinson, Charles B., D.D.S.
Baker, Dr. H. A.
Bazin, Dr. J. A.
Blair, L. P., D.D.S.
Bryan, Dr. L. C.
Bruce, James A., D.D.S.
Bulkley, T. Duncan, A.M., M.D.
Byrnes, B. S., D.D.S.
Cooke, William P., D.M.D.
Curtis, G. L , M.D., D.D.S.
Dodge, Henry N., M.D., D.D.S.
Dwinelle, W. H., M.D., D.D.S.
Fellows, Dr. D. W.
Gates, William H., D.D.S.
Gibson, Dr. Kasson C.
Hamilton, Dr. H. F.
Howe, J. Morgan, M.D., D.D.S.
Jackson, V. H., D.D.S.
Laplace, Ernest, A.M., M.D.
McCausey, Dr. George H.
Marshall, John S., M.D.
Maxfield, George A., D.D.S.
Mayr, Professor Charles.
Mills, William A., D.D.S.
Mitchell, W., D.D.S.
Osman, Dr. J. Allen.
Ottolengui, B. A. R., M.D.S.
Peck, Edward S., A.M., M.D.
Reed, Dr. J. J.
Rehfuss, W. L., D.D.S.
Roberts, Dr. W. L.
Sachs, Wilhelm, D.D.S.
Smith, C. Stoddard, D.D.S.
Symington, Johnson, M.D., F.R.S.E.
Waitt, J. E., D.M.D.
Warren, Dr. George W.
Whitefield, George, M.D., D.D.S.
Wight, J. S., M.D.
Wilson, George A., D.D.S.
Winkler, George H., M.D., D.D.S.
Wright, E. P., D.D.S.

